# Supplementary material for: Solid-Phase Synthesis of the Bicyclic Peptide OL-CTOP Containing Two Disulfide Bridges, and an Assessment of Its In Vivo μ-Opioid Receptor Antagonism after Nasal Administration
Source: Molecules. 2023 Feb 15;28(4):1822. doi: 10.3390/molecules28041822 (PMC9963138; doi:10.3390/molecules28041822)
Supplement: Supplementary file 1 [file molecules-28-01822-s001.zip › molecules-2085109-supplementary.pdf]

Supplementary Materials

Solid-phase synthesis of a bicyclic peptide OL-CTOP containing two disulfide bridges and assessment of its *in vivo* μ-opioid receptor antagonism after nasal administration

Ramanjaneyulu Rayala[1ǂ], Annika Tiller[1], Shahayra A. Majumder[2], Heather M. Stacy[2], Shainnel O. Eans[2], Aleksandra Nedovic[1,] Jay P. McLaughlin[2*] and Predrag Cudic[1*]

[1]Department of Chemistry and Biochemistry, Charles E. Schmidt College of Science, Florida Atlantic University, 777 Glades Road, Boca Raton, FL 33431, USA

[2]Department of Pharmacodynamics, School of Pharmacy, University of Florida, 1345 Center Drive, Gainesville, FL 32610, USA

ǂ Current address: Center for Translational Science, Florida International University, 11350 SW Village Pakway Port St. Lucie, FL 34987, USA

*Correspondence: jmclaughlin@cop.ufl.edu; +1-352-273-7207 (J.P.M.);
pcudic@fau.edu; +1-561-799-8375 (P.C.)

RP-HPLC analysis: C18 monomeric column (Grace Vydac, 250 × 4.6 mm, 5 mm, 120 Å), 1 ml/min flow rate, and elution method with a linear gradient of 2 → 100% B over 45 min, where A is 0.1% TFA in H_2O, and B is 0.08% TFA in CH_3CN. Because of limited solubility in water, all peptides were dissolved in DMSO and DMSO solutions were used for RP-HPLC analysis.

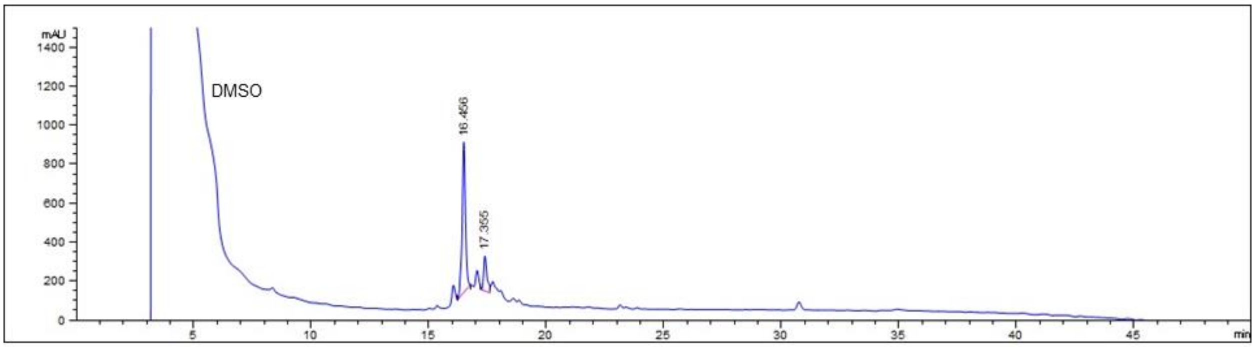

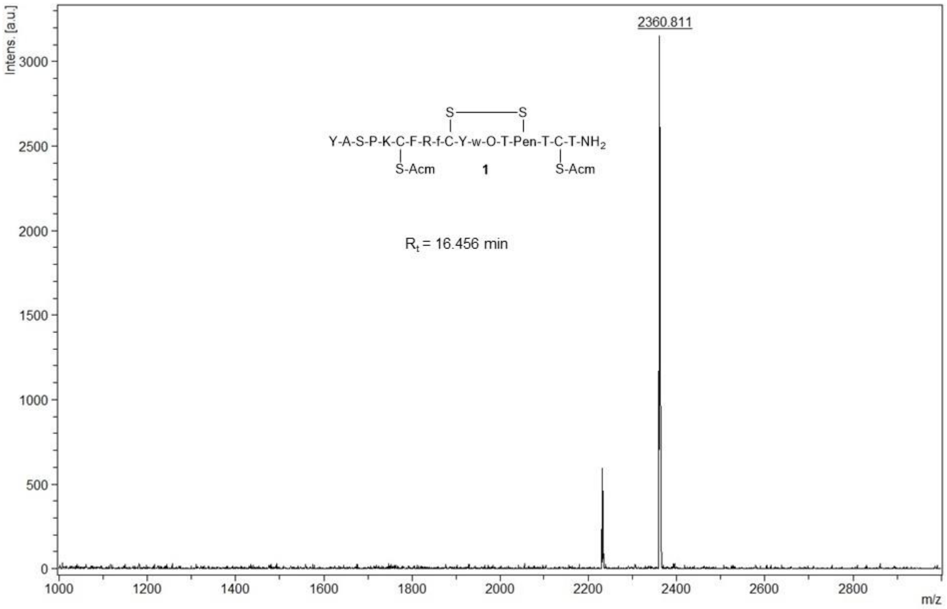

Expected $[M+H]^+$= 2360.036 Da, observed $[M+H]^+$ = 2360.811 Da

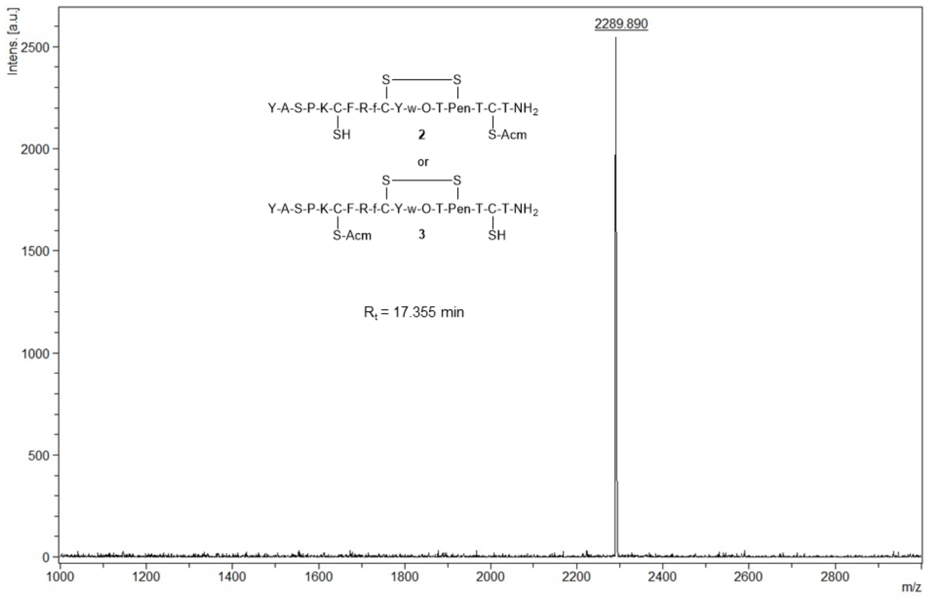

Expected [M+H]+ = 2288.99 Da, observed [M+H]+ = 2289.89 Da

Figure S1. HPLC traces and MALDI ToF MS spectra of synthetic precursors following Cys[10] and Pen[15] oxidation.

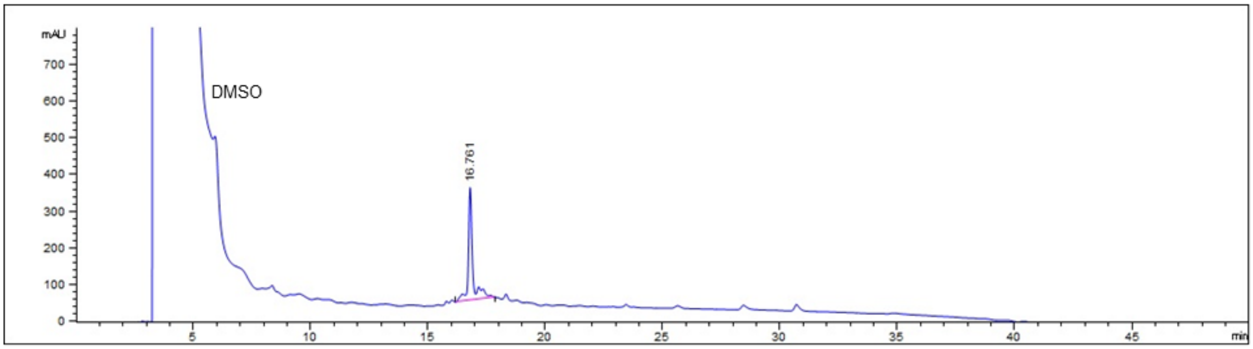

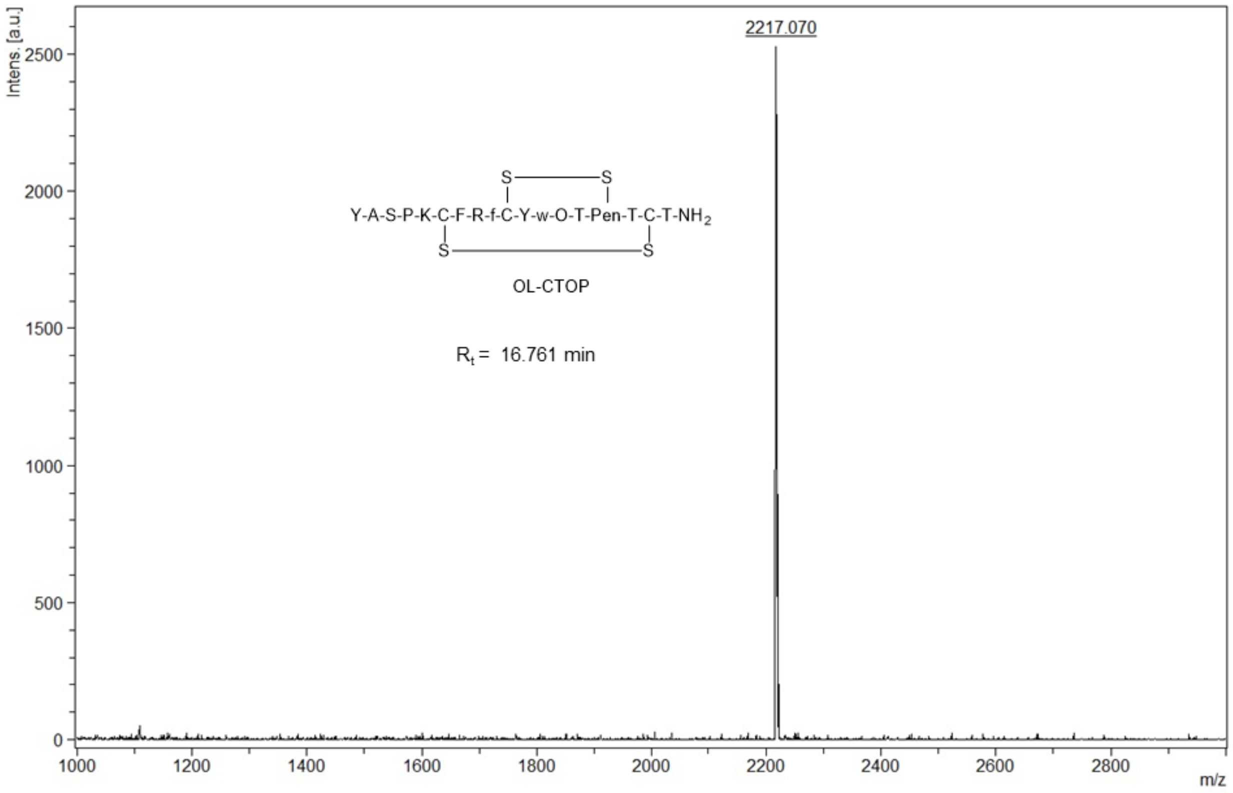

Expected $[M+H]^+$ = 2215.946 Da, observed $[M+H]^+$ = 2217.070 Da

Figure S2. HPLC traces and MALDI ToF MS spectra of synthetic precursors following Cys[6] and Cys[17] oxidation.

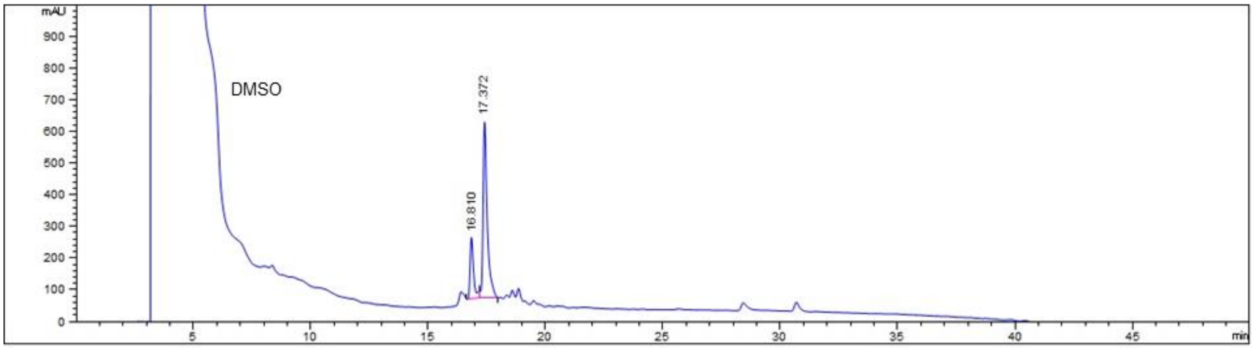

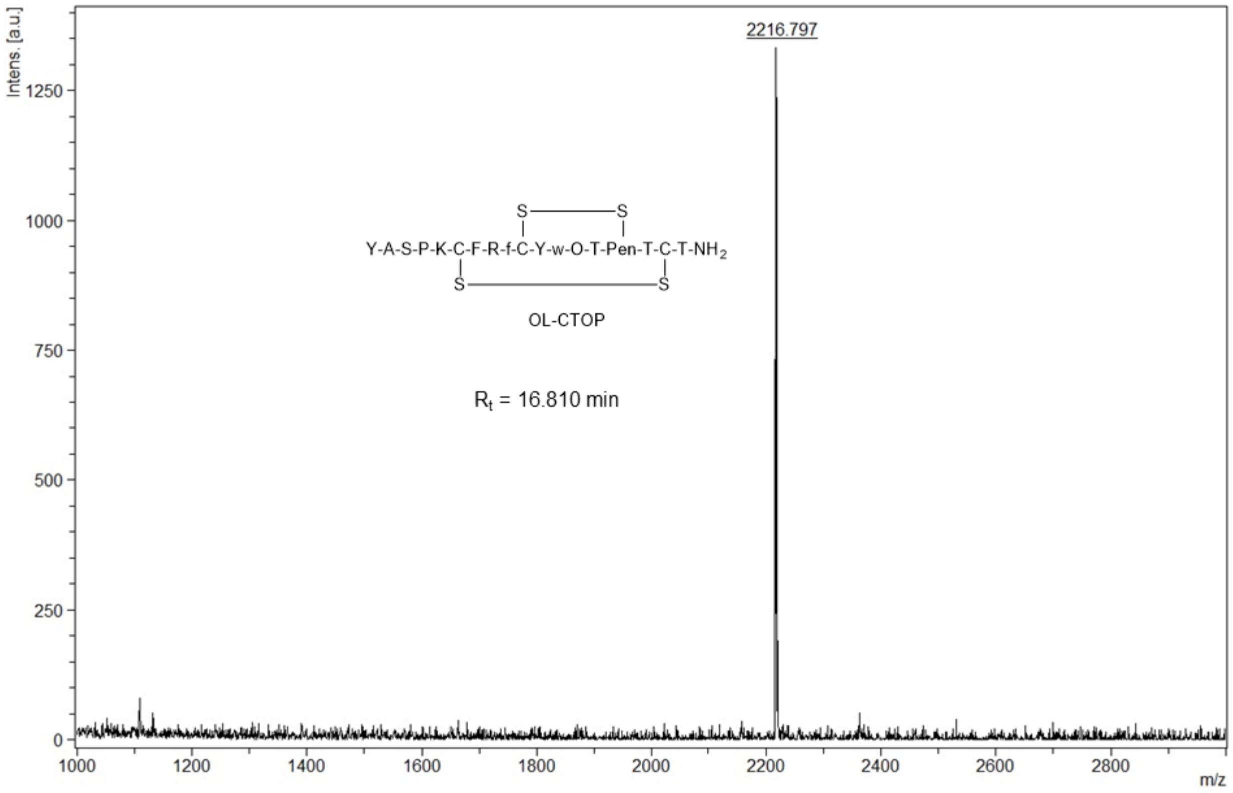

Expected $[M+H]^+$ = 2215.946 Da, observed $[M+H]^+$ = 2216.797 Da

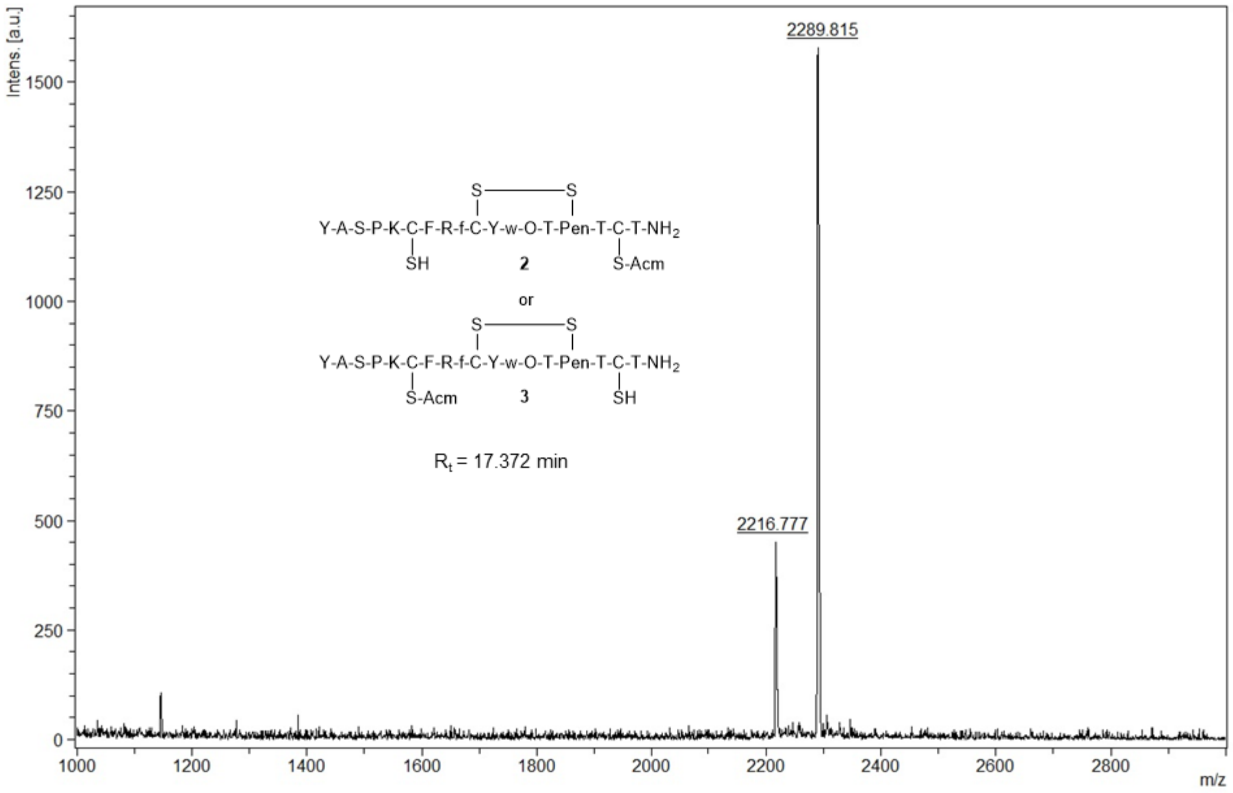

Expected [M+H]+ = 2288.999 Da, observed [M+H]+ = 2289.815 Da

Figure S3. HPLC traces and MALDI ToF MS spectra of OL-CTOP following single-step oxidation.

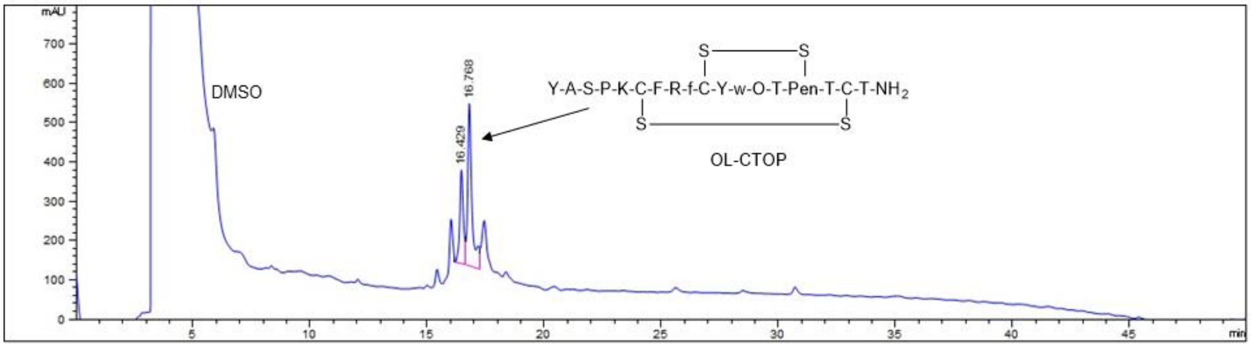

Figure S4. Representative HPLC trace of crude OL-CTOP following single-step oxidation with 30 eq of DMSO as a co-solvent.

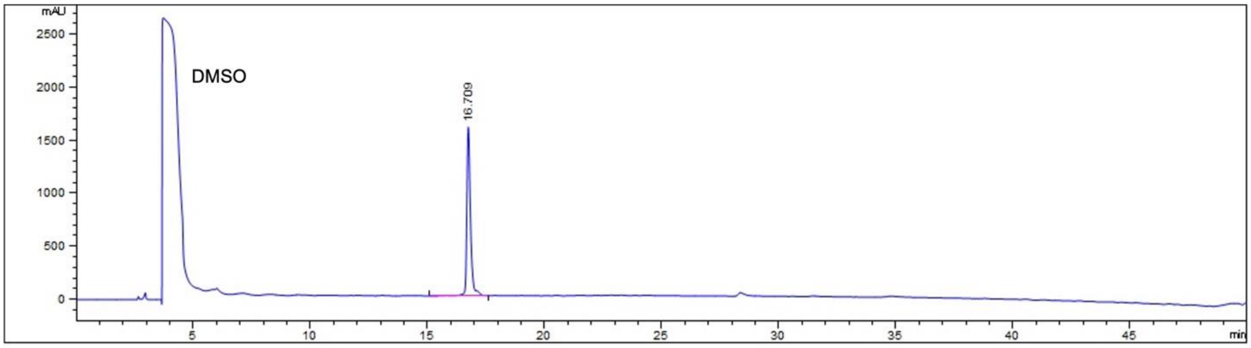

Figure S5. HPLC trace of OL-CTOP following trypsin digestion, 0 min time interval.

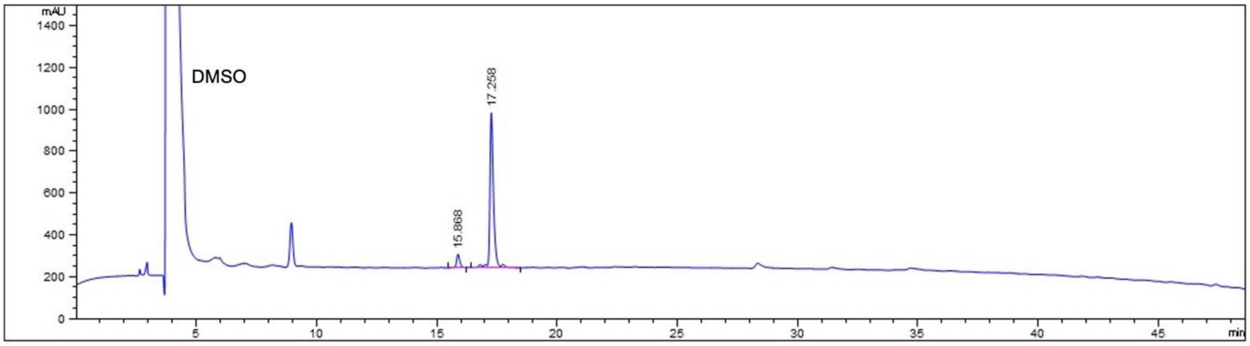

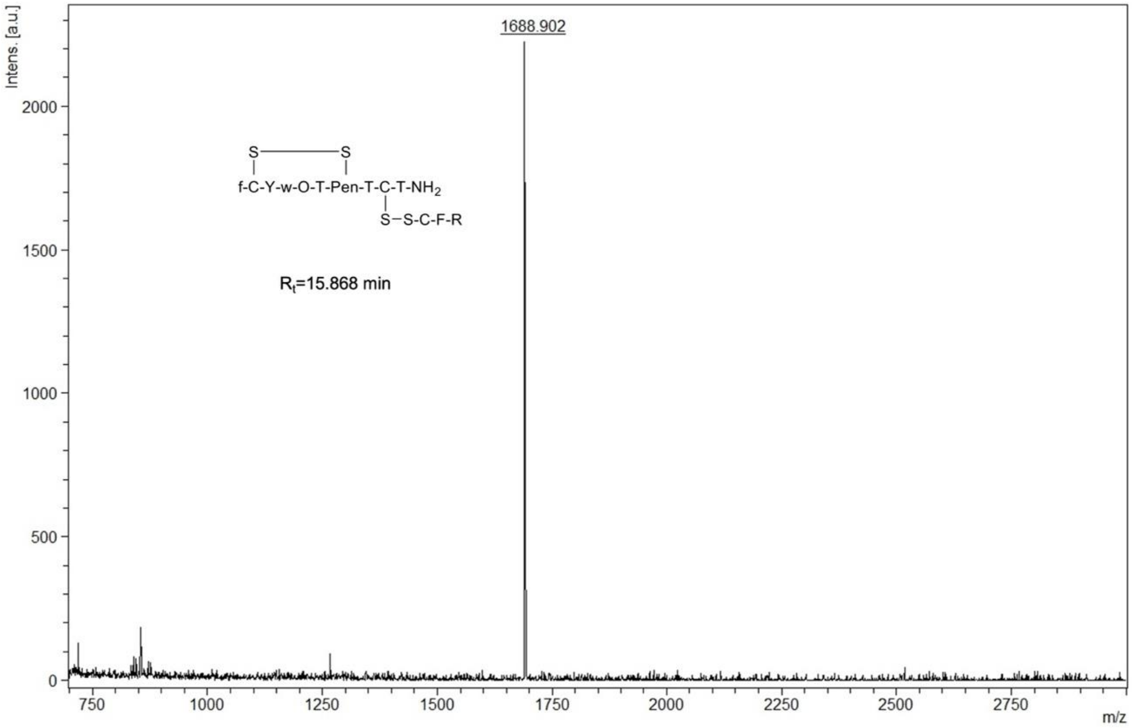

Expected $[M+H]^+$= 1687.68 Da, observed $[M+H]^+$ = 1688.902 Da

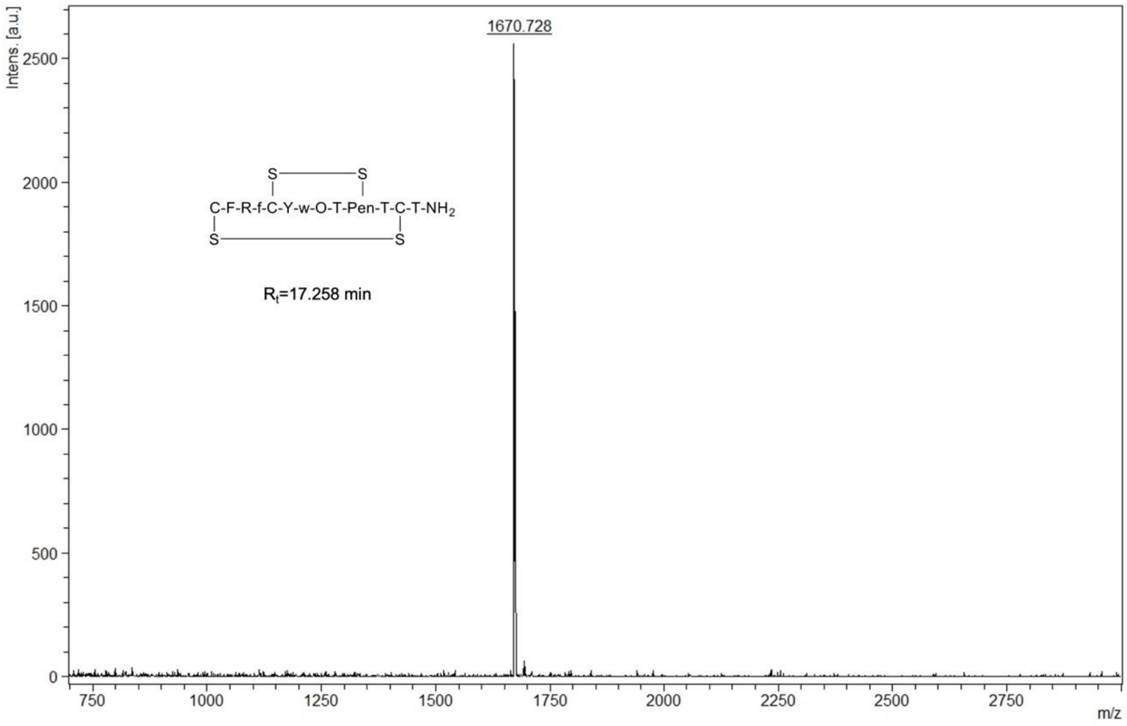

Expected [M+H]+= 1671.01 Da, observed [M+H]+ = 1670.728 Da

Figure S6. HPLC trace and MALDI ToF MS spectra of OL-CTOP following trypsin digestion, 10 min time interval.

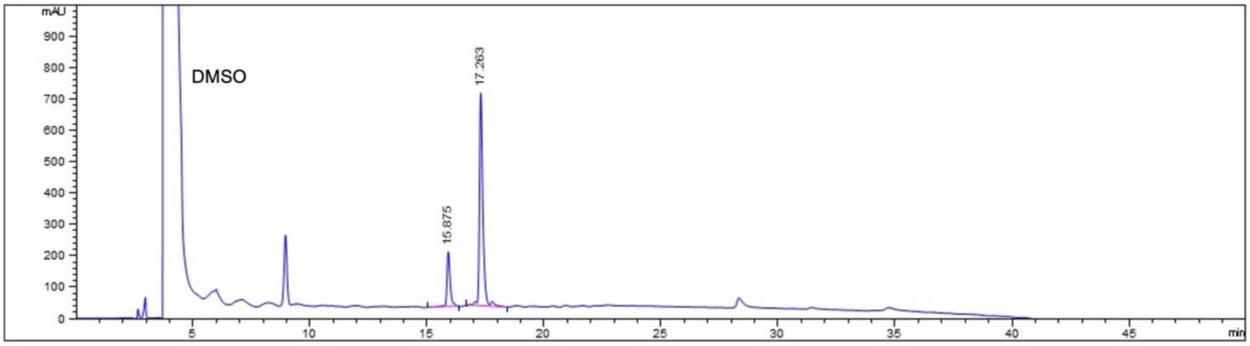

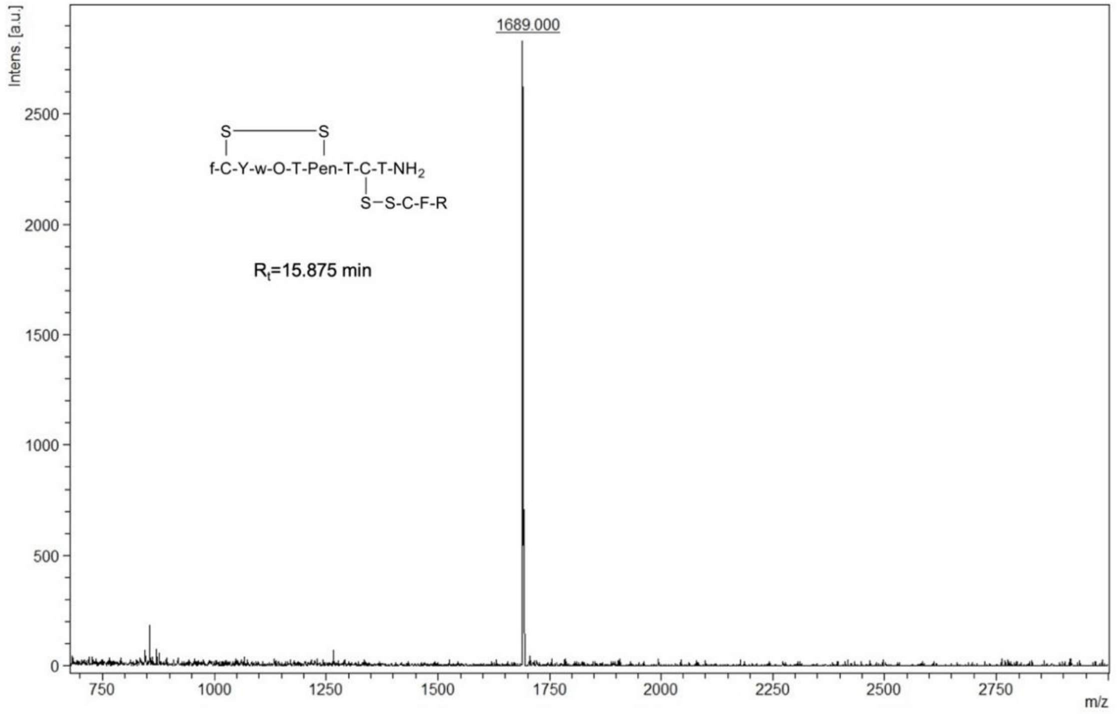

Expected [M+H]+= 1687.68 Da, observed [M+H]+ = 1689.000 Da

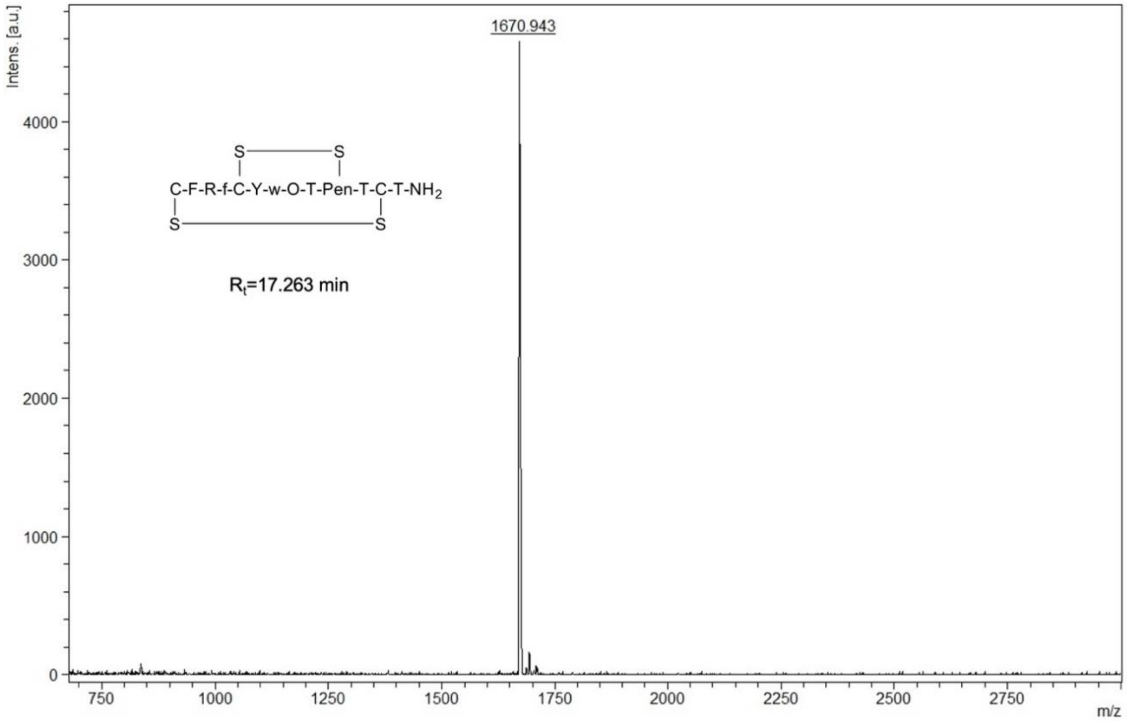

Expected [M+H]+= 1671.01 Da, observed [M+H]+ = 1670.943 Da

Figure S7. HPLC trace and MALDI ToF MS spectra of OL-CTOP following trypsin digestion, 1h time interval.

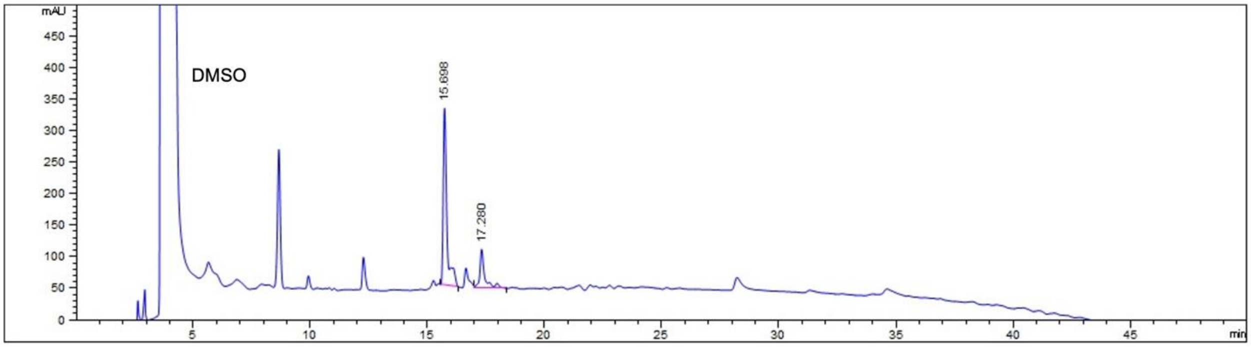

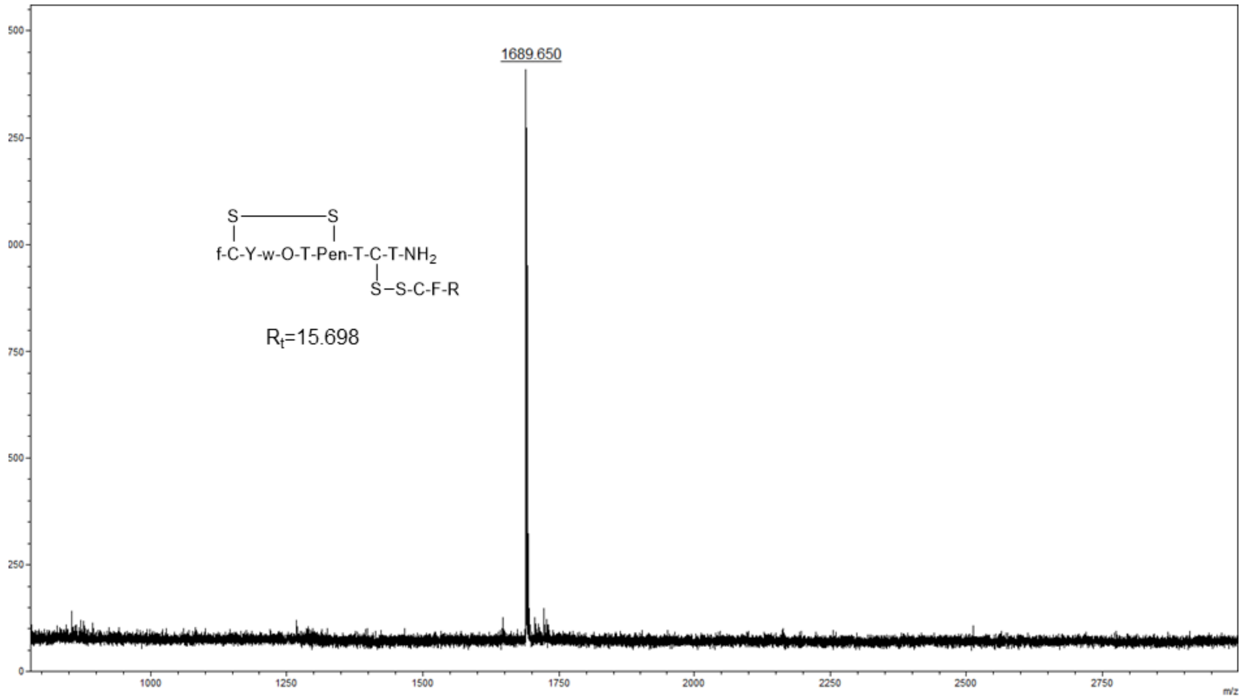

Expected $[M+H]^+$= 1687.68 Da, observed $[M+H]^+$ = 1689.650 Da

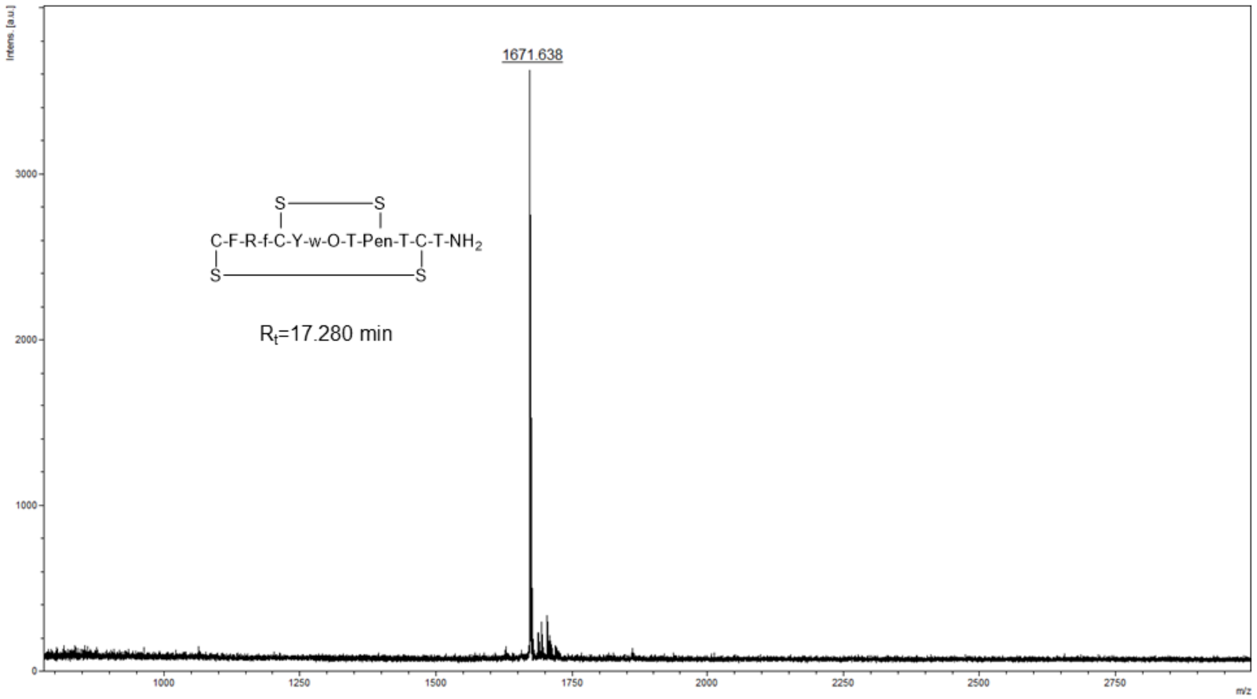

Expected $[M+H]^+$=1669.67 Da, observed $[M+H]^+$ = 1671.638 Da

Figure S8. HPLC trace and MALDI ToF MS spectra of OL-CTOP following trypsin digestion, 24h time interval.
